# The diagnostic pathway of Parkinson’s disease: a cross-sectional survey study of factors influencing patient dissatisfaction

**DOI:** 10.1186/s12875-017-0652-y

**Published:** 2017-08-25

**Authors:** Annette O. A. Plouvier, Tim C. Olde Hartman, Olga A. de Bont, Sjoerd Maandag, Bastiaan R. Bloem, Chris van Weel, Antoine L. M. Lagro-Janssen

**Affiliations:** 1 Department of Primary and Community Care, Radboud university medical center, Postbus 9101, 6500 HB Nijmegen, The Netherlands; 2 Department of Neurology and Parkinson Center Nijmegen, Donders Institute for Brain, Cognition and Behaviour, Radboud university medical center, Nijmegen, The Netherlands; 3 Department Health Services Research and Policy, Australian National University, Canberra, Australia

**Keywords:** Parkinson’s disease, Patient dissatisfaction, Diagnostic pathway, Second opinion, Experienced delay, Primary care

## Abstract

**Background:**

The diagnostic pathway of Parkinson’s disease (PD) is often complicated. Experiences during this pathway can affect patients’ satisfaction and their confidence and trust in healthcare providers. Although healthcare providers cannot influence the impact of the diagnosis, they can influence how patients experience the pathway. This study, therefore, aims to provide insight into PD patients’ dissatisfaction with the diagnostic pathway and to describe the factors that influence it.

**Methods:**

We carried out a cross-sectional survey study among 902 patient members of the Dutch Parkinson’s Disease Association, who were each asked to write an essay about their diagnostic pathway. A coding format was developed to examine the content of these essays. Inter-observer agreement on coding patient dissatisfaction was calculated using Cohen’s kappa. The χ^2^ test and a multivariable logistic regression analysis were performed to assess the relation between dissatisfaction and sex, level of education, duration of the pathway, communication with the general practitioner (GP) and the neurologist, the number of healthcare providers involved, whether or not a second opinion had taken place (including the person who initiated it) and diagnostic delay (taking into consideration who caused the delay according to the patient). A subgroup analysis was performed to gain insight into sex-related differences.

**Results:**

Of all patients, 16.4% explicitly described they were dissatisfied with the diagnostic pathway, whereas 4.8% were very satisfied. The inter-observer agreement on coding dissatisfaction was κ = 0.82. The chance of dissatisfaction increased with a lower level of education, the involvement of more than one additional healthcare provider, a second opinion initiated by the patient and delay caused by a healthcare provider. When only the GP and the neurologist were involved, women were more likely to be dissatisfied than men.

**Conclusions:**

PD patients’ dissatisfaction with the diagnostic pathway is related to a lower level of education, a second opinion initiated by the patient and experienced diagnostic delay. GPs can positively influence patients’ experiences if they are aware of these risk factors for dissatisfaction and pay extra attention to communication and shared decision making. This will contribute to a trusting therapeutic relationship that is indispensable with progression of the disease.

**Electronic supplementary material:**

The online version of this article (doi:10.1186/s12875-017-0652-y) contains supplementary material, which is available to authorized users.

## Background

Patients’ experiences during the pathway to a diagnosis can be negative and have long-term consequences. Research in cancer patients shows that patients tend to lose confidence and trust in their general practitioner (GP) if the nature of the presented symptoms is not immediately recognised by the GP and if multiple visits to the GP are necessary before referral takes place [1–3]. Moreover, patients may interpret the need to consult their GP repeatedly as lack of responsiveness and thus as delay caused by the GP, resulting in patient dissatisfaction [4].

Diagnosis timing issues may induce patients to change general practice [5]. The importance of timeliness of diagnosis related to patient dissatisfaction emerges from a study amongst cancer patients, showing that patients suffering from cancer types that are difficult to recognise, such as ovarian cancer and multiple myeloma, are more likely to change practice than patients with types of cancer that are easier to recognise, such as melanoma and breast cancer [5]. In addition, long-term care experiences of cancer patients are worse for those who visited their GP several times before they were referred than for those who were referred instantly [2].

Parkinson’s disease (PD) is a progressive neurodegenerative disorder that can be difficult to diagnose [6]. Classic symptoms such as muscular rigidity and tremor are not always present and may be preceded by a variety of motor and non-motor symptoms that are not necessarily disease-specific [7–10]. When patients consult their GP, symptoms are often still limited and embedded in clinical uncertainty, while referral to the neurologist takes place later in the disease trajectory [10–12]. This may explain the difficulties GPs encounter in recognising PD as the common cause of these symptoms and in referring accordingly [10, 12]. As a consequence, the pathway to the diagnosis of PD can be lengthy and uncertain and, unless well explained, it is reasonable to expect a negative influence on patient confidence, trust and satisfaction [6, 13].

Although the impact of a PD diagnosis cannot be taken away completely, healthcare providers can have an influence on how patients experience the diagnostic pathway. It is known that PD patients’ dissatisfaction with the way the diagnosis of PD is explained to them has an impact on health-related quality of life [14]. Lack of involvement in therapy decisions is also negatively related to satisfaction and compliance with therapy [15]. However, research into patient experiences of the diagnostic pathway of PD is limited and does not provide any insight into factors influencing patient dissatisfaction [13, 16]. Patients will benefit from a sustained trusting relationship with their GPs, in which they have confidence in the personal care provided by the GP, as progression of the disease will inevitably cause health problems that require the GP’s involvement [17]. In order to optimise patients’ experiences of the pathway to the diagnosis of PD and, hence, to contribute to a trusting patient-doctor relationship, this study aims to improve our understanding of PD patients’ dissatisfaction with the diagnostic pathway and to describe the factors influencing it.

## Methods

### Recruitment and data collection

We conducted a cross-sectional survey study among patient members of the Dutch Parkinson’s Disease Association. All members with a known email address (*n* = 4717) were approached digitally to enlist their participation. Patients were asked to fill in their demographic characteristics at the time of diagnosis: sex, age, highest level of education finished, employment status and civil status. They were also asked to describe their experiences of the pathway from the first recognisable symptom(s) to the diagnosis of PD. To facilitate patients in formulating their essay, we provided them with a number of guiding questions that were based on the literature and expert opinion and had been tested in a pilot study (Table 1). In case patients had questions, concerns or hesitations, they could contact the researcher (AP). After finishing their essays, patients had to agree to submission, a step that was assessed as informed consent. Participation was one-time only, voluntarily and anonymous. The research ethics committee of the Radboud university medical center examined the protocol of the study and concluded that the study could be carried out in the Netherlands without needing approval by the regional research ethics committee.

**Table 1:** Questions guiding patients to describe their experiences of the diagnostic pathway of Parkinson’s disease

Question	
1.	Can you describe the first symptom(s) that eventually turned out to be a forerunner sign of PD? What did you do when you experienced this symptom or these symptoms?
2.	Can you describe what happened next, until the moment you were diagnosed with PD?
3.	What role was there for people in your surroundings during the diagnostic pathway?
4.	Looking back on the diagnostic pathway, how do you feel about the timing of the diagnosis? Can you describe the consequences of this timing for you and your family?

A qualitative analysis of a purposive sample of 52 essays preceded this study. Purposive sampling was based on the collected demographic characteristics at the time of diagnosis. We refer to the paper describing the qualitative analysis for more detailed information on recruitment, data collection and results [16]. The qualitative analysis results were used to create a format to examine the content of all essays. Details on the coding format are described in Additional file 1.

Though patient dissatisfaction with the diagnostic pathway was the main focus of the current study, patients were not explicitly asked for their satisfaction or dissatisfaction. Rather, we encouraged them to describe their feelings about the timing of the diagnosis and the consequences of this timing in order to gain insight into patients’ spontaneous reporting of the diagnostic pathway (Table 1). We only applied codes if patients spontaneously and unmistakably expressed their satisfaction or dissatisfaction: ‘satisfied’ was coded when patients were clearly positive, and ‘dissatisfied’ was coded when patients explicitly mentioned problems or made negative remarks. All other cases, including the expression of mixed emotions, were coded ‘neutral’. To enable the researchers to interpret satisfaction and dissatisfaction with the diagnostic pathway, it was defined as ‘the overall feeling a patient expressed about the diagnostic pathway in his/her essay’, and it was independently coded by two researchers (AP, OdB) in a random sample of 225 essays (25%) to enable calculation of inter-observer agreement.

The same researchers also independently coded 154 essays (17.1%) completely, initially to create consensus on the coding method, and later to discuss doubts in coding. The other 748 essays were coded by one researcher (OdB). Codes were only applied if patients in their essay explicitly described the duration of the pathway, communication with the GP or the neurologist, the number of different healthcare providers involved, a second opinion or experienced delay.

### Data analysis

Statistical analyses were conducted using Statistical Package for the Social Sciences (SPSS) 22.0. Descriptive statistics were calculated. As patient dissatisfaction was the main focus of our analysis, the expressed feelings were divided into two categories: dissatisfied and neutral/satisfied. Cohen’s kappa was used to calculate inter-observer agreement.

We wanted to assess the relation between dissatisfaction with the diagnostic pathway and a selection of factors. These factors were selected based on literature [5, 18], the results of the preceding qualitative analysis [16] and expert opinion. The χ^2^ test was used to assess the relation between dissatisfaction and the demographic variables sex and level of education, the latter divided into low (primary school/vocational education), medium (secondary school) and high (higher professional education/university). Moreover, we assessed the relation between dissatisfaction and duration of the diagnostic pathway (divided into unknown, <2 years or ≥2 years on the basis of the literature [6, 8, 9]); communication with the GP or the neurologist (negative, neutral/positive); and the number of different healthcare providers involved (0, 1, 2, ≥3). As guidelines in the Netherlands describe the involvement of a GP and a neurologist as usual care in the pathway to the diagnosis of PD, these healthcare providers were excluded from the number of healthcare providers involved [11, 19].

In addition, we performed the χ^2^ test to assess the relation between dissatisfaction and second opinion. Second opinion was defined as ‘the involvement of a second neurologist during the pathway towards the diagnosis of PD’ and was categorised into: no second opinion/not mentioned; second opinion on the patient’s initiative (including the combined initiative of patient and healthcare provider); and second opinion on the healthcare provider’s initiative. We also assessed the relation between dissatisfaction and experienced diagnostic delay, taking into consideration who caused the delay according to the patient. Delay was divided into: no delay; not (clearly) mentioned; caused by the patient (including caused by both the patient and the healthcare provider); caused by the healthcare provider(s); and unknown who caused it.

A multivariable logistic regression analysis was performed to assess the independent association between dissatisfaction and sex, level of education, duration of the diagnostic pathway, the number of different healthcare providers involved, second opinion and experienced delay. As only few patients explicitly described their communication with the GP and the neurologist, this factor was excluded from the regression analysis. We also excluded second opinions if it was unknown on whose initiative they had taken place. As the literature shows that female patients tend to be more dissatisfied with care than male patients [18], we also performed a subgroup analysis to gain insight into possible sex-related differences. Therefore, we added interaction terms of sex with the other variables to the multivariable regression model. A *P-*value less than 0.05 was considered statistically significant.

## Results

### Characteristics of the study population

**Fig. 1:**
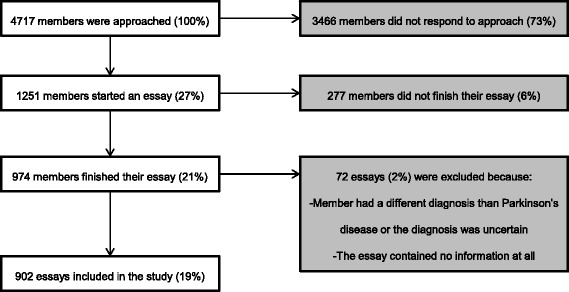
From recruitment of patient members of the Dutch Parkinson’s Disease Association to essays included

Of all patient members who received an email, 27% started and 21% finished the essay. Seventy-two essays were excluded due to incorrect or uncertain diagnosis of PD or a complete lack of information. Finally, 902 essays were included in this study (Fig. 1). More men than women participated, and most patients had a high level of education (Table 2). Mean age at the time of diagnosis was 60 years (Standard Deviation (SD) 9.9).

**Table 2:** Included PD patients’ characteristics at the time of diagnosis

Demographic variable		*n =* 902 (100%)
Sex	Male	550 (61.0%)
	Female	352 (39.0%)
Mean age in years (SD)	60.1 (9.9)	
Level of education	Low	250 (27.7%)
	Medium	284 (31.5%)
	High	368 (40.8%)
Employment status	Employed	352 (39.0%)
	Self-employed	71 (7.9%)
	Retired	307 (34.0%)
	Recipient of sickness benefits	18 (2.0%)
	Unemployed	86 (9.5%)
	Combination of employments/other	68 (7.5%)
Civil status	Single ^a^	80 (8.9%)
	With partner	596 (66.1%)
	With family (including partner)	217 (24.1%)
	Other	9 (1.0%)

^a^Including widowed and divorced

### Patient dissatisfaction with the diagnostic pathway

The inter-observer agreement of the two researchers (AP, OdB) on coding patient dissatisfaction with the diagnostic pathway was κ = 0.82 (95% Confidence Interval (CI) 0.72–0.91).

More than one in seven patients (*n* = 148; 16.4%) explicitly described they were dissatisfied with the experienced diagnostic pathway. Most patients (*n* = 711; 78.8%) did not clearly express their opinion, while less than 5 % (*n* = 43; 4.8%) mentioned they were satisfied.

Dissatisfaction was significantly associated with several factors. Female sex (*P* < 0.01), duration of the pathway (*P* < 0.001), communication with the GP or the neurologist (*P* < 0.001 and *P* < 0.01, respectively), the number of healthcare providers involved (*P* < 0.001), second opinion (*P* < 0.001) and experienced delay (*P* < 0.001) increased the chance of dissatisfaction (Table 3). Of the patients who felt the healthcare provider caused delay, more than half were dissatisfied (*n* = 111; 61.0%), whereas less than 5% of the patients, who did not experience delay or felt delay was due to themselves, were dissatisfied (*n* = 15; 3.2% and *n* = 2; 2.6%, respectively).

**Table 3:** Univariable analysis of factors influencing patient dissatisfaction with the diagnostic pathway of Parkinson’s disease

Variable	Patient dissatisfaction *n* (%)	Patient satisfaction/neutral *n* (%)	*P* value
Sex (*n* = 902)			<0.01*
Male	74 (13.5%)	476 (86.5%)	
Female	74 (21.0%)	278 (79.9%)	
Level of education (*n* = 902)			0.22
Low	49 (19.6%)	201 (80.4%)	
Medium	40 (14.1%)	244 (85.9%)	
High	59 (16.0%)	309 (84.0%)	
Duration of the diagnostic pathway (*n* = 902)			<0.001*
Unknown	54 (13.3%)	352 (86.7%)	
< 2 years	28 (12.1%)	204 (87.9%)	
≥ 2 years	66 (25.0%)	198 (75.0%)	
Communication with the general practitioner (*n* = 77)			<0.001*
Negative	40 (69.0%)	18 (31.0%)	
Neutral/positive	2 (10.5%)	17 (89.5%)	
Communication with the neurologist (*n* = 78)			<0.01*
Negative	41 (62.1%)	25 (37.9%)	
Neutral/positive	1 (8.3%)	11 (91.7%)	
Number of healthcare providers involved (*n* = 902)^a^			<0.001*
0	43 (8.2%)	484 (91.8%)	
1	52 (21.3%)	192 (78.7%)	
2	31 (34.8%)	58 (65.2%)	
≥ 3	22 (52.4%)	20 (47.6%)	
Second opinion (*n* = 856)^b^			<0.001*
No/not mentioned	93 (12.5%)	650 (87.5%)	
Yes, on the patient’s initiative	29 (45.3%)	35 (54.7%)	
Yes, on the healthcare provider’s initiative	12 (24.5%)	37 (75.5%)	
Experienced delay (*n* = 902)			<0.001*
No delay	15 (3.2%)	454 (96.8%)	
Not (clearly) mentioned	9 (6.8%)	124 (93.2%)	
Yes, caused by the patient	2 (2.6%)	74 (97.4%)	
Yes, caused by a healthcare provider	111 (61.0%)	71 (39.0%)	
Yes, unknown who caused it	11 (26.2%)	31 (73.8%)	

*Statistically significant, *P* < 0.05

^a^Excluding GP and neurologist

^b^Excluding second opinion, initiative unknown

The multivariable analysis showed that low-educated patients were more likely to be dissatisfied than medium and high-educated patients (Odds Ratio (OR) 0.45; CI 0.2–0.9 and OR 0.46; CI 0.2–0.9, respectively). The chance of dissatisfaction was also significantly higher when more than one additional healthcare provider was involved. With the involvement of two extra healthcare providers, the odds ratio for patient dissatisfaction was 2.53 (CI 1.2–5.3) compared to a situation in which only the GP and the neurologist were involved. If three or more additional healthcare providers were involved, the odds ratio for dissatisfaction was even higher (OR 3.92; CI 1.4–10.7). A second opinion on the patient’s initiative increased the chance of dissatisfaction compared to cases without a second opinion (OR 5.04; CI 2.3–10.9). In addition, when patients experienced delay caused by a healthcare provider, they were significantly more likely to be dissatisfied than patients who did not experience delay (OR 38.78; CI 20.0–75.0) (Table 4).

**Table 4:** Multivariable logistic regression of factors influencing patient dissatisfaction with the diagnostic pathway of Parkinson’s disease

Variable (*n* = 856)^b^	Odds ratio (OR) for dissatisfaction	95% Confidence Interval (CI)	*P* value
Sex			0.12
Male	Reference		
Female	1.50	0.9–2.5	0.12
Level of education			0.02*
Low	Reference		
Medium	0.45	0.2–0.9	0.02*
High	0.46	0.2–0.9	0.01*
Duration of the diagnostic pathway			0.47
Unknown	Reference		
< 2 years	1.20	0.6–2.4	0.61
≥ 2 years	1.43	0.8–2.5	0.22
Number of healthcare providers involved^a^			0.01*
0	Reference		
1	1.66	0.9–3.0	0.09
2	2.53	1.2–5.3	0.01*
≥ 3	3.92	1.4–10.7	<0.01*
Second opinion			<0.001*
No/not mentioned	Reference		
Yes, on the patient’s initiative	5.04	2.3–10.9	<0.001*
Yes, on the healthcare provider’s initiative	2.11	0.8–5.4	0.12
Experienced delay			<0.001*
No delay	Reference		
Not (clearly) mentioned	1.85	0.7–4.6	0.19
Yes, caused by the patient	0.84	0.2–4.0	0.83
Yes, caused by a healthcare provider	38.78	20.0–75.0	<0.001*
Yes, unknown who caused it	7.14	2.7–19.0	<0.001*

*Statistically significant, *P* < 0.05

^a^Excluding GP and neurologist

^b^Excluding second opinion, initiative unknown

Men and women significantly differed in the relation between dissatisfaction with the diagnostic pathway and the number of healthcare providers involved during this pathway. If only the GP and neurologist were involved, female patients were more likely to be dissatisfied than male patients (OR 3.11; CI 1.4–7.0). There were no significant differences between men and women in the chance of dissatisfaction with the involvement of one or more additional healthcare provider(s) (OR 0.58; CI 0.2–1.4 with one, OR 1.63; CI 0.4–5.8 with two, and OR 1.92; CI 0.3–12.1 with three or more healthcare providers involved). No interaction effects were found for any other variables (results provided in Additional file 2).

## Discussion

Most patients in our study do not describe dissatisfaction with the diagnostic pathway of PD. However, more than one in seven patients is explicitly dissatisfied. The chance of dissatisfaction is increased with a lower level of education, the involvement of more than one additional healthcare provider, a second opinion on the patient’s initiative and delay caused by the healthcare provider. In addition, if only the GP and neurologist have been involved in the diagnostic pathway, women are more likely to be dissatisfied than men.

Although dissatisfaction with the diagnostic pathway of PD appears to be playing a limited role in quantitative terms, dissatisfaction with the initial diagnostic process can have an impact on long-term care, stressing the importance of paying attention to it: cognitions formed at an early stage tend to determine care experiences during the further treatment episode [2, 5, 20].

Our study shows that it is not the duration of the diagnostic pathway of PD on its own that leads to patient dissatisfaction, but that other factors appear to be important as well. In line with the literature, the women in our study are more likely to be dissatisfied than the men [5, 18]. Contrary to what we expected, however, the chance of dissatisfaction is highest for low-educated patients [5, 18]. As only few patients in our study explicitly mention their communicative experiences with the GP and the neurologist, we could not demonstrate an independent association between dissatisfaction and communication. It is likely, though, that communication at least partly explains the finding that low-educated patients are more often dissatisfied than patients with higher education as low-educated PD patients are known to have a lower level of health literacy than high-educated patients and a low level of health literacy negatively influences patients’ ability to obtain and understand information about a disease [21, 22].

In the case of PD, the complexity and abstractness of the process in the brain that causes the complaints and the varying expression of the disease can be difficult for a doctor to explain understandably and challenging for a patient to grasp fully. An earlier study shows that the way the diagnosis of PD is communicated to a patient is very important [13]. Moreover, the difficulty for GPs to recognise PD and the fact that a diagnosis is not 100% certain until autopsy is performed are likely to influence patients’ experiences of the diagnostic pathway of PD as well [10, 17]. Cancer patients, for example, mention they feel uncertain and anxious if referral is not explained carefully, and patients with amyotrophic lateral sclerosis report falsely raised hopes after every negative investigation [20, 23]. Our study shows that patients who feel that their healthcare provider is responsible for delay in the diagnostic pathway are far more likely to be dissatisfied than patients who do not describe delay.

Lack of communication during the diagnostic pathway is also known to have a long-term impact on the patient-doctor relationship, a relationship that depends on patients’ satisfaction and their confidence and trust in the healthcare provider - with the risk of disappointment as the distinguishing feature between the latter two: in a situation of trust one is more likely to be disappointed- [20, 24]. In our study, patient dissatisfaction is related to a second opinion on the patient’s initiative. Although we do not know whether dissatisfaction is the cause or the result of the patient’s request for a second opinion, earlier research shows that dissatisfaction is negatively associated with trust and that trust limits the tendency for patients to request for a second opinion [24]. Physicians who listen carefully, behave empathically and communicate clearly are more likely to be trusted by their patients [24, 25]. Moreover, trust is further enhanced if patients feel they are treated as equal partners [24, 25].

Though the negative impact of being diagnosed with PD cannot be ruled out, GPs can contribute to their patients’ experiences of the diagnostic pathway of PD in a positive way by using their central role in symptom recognition and referral to communicate openly about the clinical uncertainty involved in PD diagnosis and about the expectations of referral, while taking into account a patient’s level of health literacy and offering scope for questions, hesitations and emotions. This is how GPs and patients can go through the diagnostic pathway of PD together and make shared decisions whenever possible or desirable. It is likely that such an experience will contribute to patient satisfaction with the pathway and will help to maintain a trusting therapeutic relationship that is indispensable with progression of the disease [17].

### Strengths and limitations of the study

To the best of our knowledge, this is the first study that reports on patient dissatisfaction with the diagnostic pathway of PD and factors that might be of influence. We included a large number of essays. Moreover, we used an original approach to mixed methods research [26]. Our data are based on patients’ spontaneous reporting, thus reflecting what matters most to them, rather than reflecting their recognition of pre-determined items.

We used a coding format that was based on the results of our preceding qualitative study, and a considerable part of all essays was independently coded by two researchers, who reached consensus in case of coding disagreement [16]. The strength of the inter-observer agreement on coding dissatisfaction can be considered ‘almost perfect’, confirming our opinion that we used a reliable method to extract the content of the essays [27]. We feel confident that the results of our study provide valuable new information that can be used to improve patient experiences of the diagnostic pathway of PD.

Nevertheless, there are limitations to consider when interpreting the results of this study. As patients described their experiences retrospectively, recall bias cannot be ruled out. In addition, this study used the diagnostic pathway of PD as a starting-point, yet patients are likely to have had previous care experiences with their GPs, and their satisfaction or dissatisfaction with these earlier care episodes may have influenced their experiences with the diagnostic pathway of PD as well as their description of it. In addition, dissatisfaction with the pathway cannot solely be interpreted as dissatisfaction with the GP, as more healthcare providers have likely been involved in the diagnostic process. The spontaneous reporting method does not allow for interpretation of causality and if information on a second opinion is lacking, for example, it may not have been performed or it may not have been reported.

For practical reasons, we chose to approach only those PD patients in the Netherlands who are members of the Dutch Parkinson’s Disease Association, and not all patients we approached finalised their essay. As information on the non-responders and the patients who did not finish their essay is lacking, selection bias cannot be ruled out. As a possible result of our decision to use a digital approach, the patients included in our study are relatively young, and it cannot be ruled out that this unequal distribution has influenced the results as well, as younger patients are generally known to be less satisfied [18].

## Conclusions

The diagnostic pathway of PD can be lengthy and uncertain, and more than one in seven PD patients is clearly dissatisfied with it. This study shows that patient dissatisfaction is related to a lower level of education, a second opinion on the patient’s initiative and delay that is caused by the healthcare provider according to the patient. GPs can positively influence their patients’ experiences if they are more aware of these risk factors for dissatisfaction and pay extra attention to open communication on the clinical uncertainties of the early symptoms of PD and on shared decision making on referral. This is likely to contribute to a trusting relationship between PD patients and their GPs, a relationship that is essential at all stages of the disease.

## Additional files

Additional file 1: Coding format to examine patients’ essay contents; only variables included in the analysis have been incorporated. (DOCX 22 kb)

Additional file 2: Multivariable logistic regression of factors influencing patient dissatisfaction with the diagnostic pathway of Parkinson’s disease, including interaction terms of sex with other variables. (DOCX 28 kb)

## Abbreviations

CI: Confidence interval
GP: General practitioner
OR: Odds ratio
PD: Parkinson’s disease
SD: Standard deviation
SPSS: Statistical package for the social sciences
