# Supplementary material for: The diagnostic pathway of Parkinson’s disease: a cross-sectional survey study of factors influencing patient dissatisfaction
Source: BMC Fam Pract. 2017 Aug 25;18:83. doi: 10.1186/s12875-017-0652-y (PMC5574149; doi:10.1186/s12875-017-0652-y)
Supplement: Supplementary file 1 — Coding format to examine patients’ essay contents; only variables included in the analysis have been incorporated. (DOCX 22 kb) [file 12875_2017_652_MOESM1_ESM.docx]

*Additional file 1. Coding format to examine patients’ essay contents; only variables included in the analysis have been incorporated*

| Variable | Values |
| --- | --- |
| Sex | 1 = Male  2 = Female |
| Year of birth | Year |
| Year of diagnosis | Year |
| Age at the time of diagnosis | Age in years |
| Highest level of education finished | Low = Primary school/Vocational education  Medium = Secondary school  High = Higher professional education/university |
| Employment status at the time of diagnosis | 1 = Employed  2 = Self-employed  3 = Retired  4 = Recipient of sickness benefits  5 = Unemployed  99 = Combination of employments/other |
| Civil status at the time of diagnosis | 1 = Single (including widowed and divorced)  2 = With partner  3 = With family (including partner)  99 = Other |
| Duration of the diagnostic pathway | 0 = unknown  1 < 2 years  2 ≥ 2 years |
| Communication with the GP during the diagnostic pathway | 0 = Not mentioned/unknown  1 = Negative  2 = Neutral/Positive |
| Communication with the neurologist during the diagnostic pathway | 0 = Not mentioned/unknown  1 = Negative  2 = Neutral/Positive |
| Healthcare providers involved in the diagnostic pathway | *Each healthcare provider as a separate variable*  0 = Not involved/not mentioned  1 = Involved |
| Second opinion | 0 = No/not mentioned  1 = Yes, on the initiative of the patient or patient and healthcare provider(s)  2 = Yes on the initiative of healthcare provider(s)  99 = Yes, initiative unknown |
| Experienced delay during the diagnostic pathway | 0 = No  1 = Not (clearly) mentioned  2 = Yes, caused by the patient or by both patient and healthcare provider(s)  3 = Yes, caused by healthcare provider(s)  4 = Yes, unknown who caused it |
| Satisfaction with the diagnostic pathway | 1 = Explicitly dissatisfied  2 = Neutral  3 = Explicitly satisfied |
