# Supplementary material for: The diagnostic pathway of Parkinson’s disease: a cross-sectional survey study of factors influencing patient dissatisfaction
Source: BMC Fam Pract. 2017 Aug 25;18:83. doi: 10.1186/s12875-017-0652-y (PMC5574149; doi:10.1186/s12875-017-0652-y)
Supplement: Supplementary file 2 — Multivariable logistic regression of factors influencing patient dissatisfaction with the diagnostic pathway of Parkinson’s disease, including interaction terms of sex with other variables. (DOCX 28 kb) [file 12875_2017_652_MOESM2_ESM.docx]

###### Additional file 2. Multivariable logistic regression of factors influencing patient dissatisfaction with the diagnostic pathway of Parkinson’s disease, including interaction terms of sex with other variables

| Variable (*n* = 856)^b^ | Odds ratio (OR) for dissatisfaction | 95% Confidence Interval (CI) | *P* value |
| --- | --- | --- | --- |
| Sex   - Male - Female | Reference  2.63 | 0.6-11.6 | 0.20  0.20 |
| Level of education   - Low - Medium - High | Reference  0.41  0.34 | 0.2-1.1  0.1-0.9 | 0.07  0.08  0.02* |
| Duration of the diagnostic pathway   - Unknown - <2 years - ≥ 2 years | Reference  1.17  1.13 | 0.4-3.2  0.5-2.5 | 0.94  0.76  0.77 |
| Number of healthcare providers involved^a^   - 0 - 1 - 2 - ≥3 | Reference  3.86  3.59  5.40 | 1.7-9.0  1.3-9.8  1.3-21.9 | <0.01*  <0.01*  0.01*  0.02* |
| Second opinion   - No/not mentioned - Yes, on the patient’s initiative - Yes, on the healthcare provider’s initiative | Reference  7.44  3.72 | 2.3-24.5  1.0-13.8 | <0.01*  <0.01*  <0.05* |
| Experienced delay   - No delay - Not (clearly) mentioned - Yes, caused by the patient - Yes, caused by a healthcare provider - Yes, unknown who caused it | Reference  2.15  1.49  50.78  1.74 | 0.6-8.2  0.2-13.2  19.4-133.0  0.2-16.4 | <0.001*  0.26  0.72  <0.001*  0.63 |
| Level of Education x Sex   - Low x Male - Medium x Male - High x Male | Reference  0.95  1.74 | 0.2-3.8  0.5-6.4 | 0.59  0.94  0.40 |
| Duration of the diagnostic pathway x Sex   - Unknown x Male - <2 years x Male - ≥ 2 years x Male | Reference  1.13  1.99 | 0.3-4.8  0.6-6.6 | 0.51  0.87  0.26 |
| **Number of healthcare providers involved^a^ x Sex**   - 0 x Male - 1 x Male - 2 x Male - ≥3 x Male | Reference  0.16  0.40  0.51 | 0.0-0.6  0.1-2.0  0.1-4.0 | **0.04***  <0.01*  0.26  0.52 |
| Second opinion x Sex   - No/not mentioned x Male - Yes, on the patient’s initiative x Male - Yes, on the healthcare provider’s initiative x Male | Reference  0.56  0.10 | 0.1-2.8  0.0-1.4 | 0.22  0.48  0.18 |
| Experienced delay x Sex   - No delay x Male - Not (clearly) mentioned x Male - Yes, caused by the patient x Male - Yes, caused by a healthcare provider x Male - Yes, unknown who caused it x Male | Reference  0.89  0.41  0.79  9.25 | 0.1-5.8  0.0-9.6  0.2-3.2  0.7-125.8 | 0.38  0.90  0.58  0.74  0.10 |

*Statistically significant, P <0.05

^a^Excluding GP and neurologist

^b^Excluding second opinion, initiative unknown

x Sex: interaction term of sex with variable (male = reference group)

**Number of healthcare providers involved^b^ x Sex** is significant, meaning that there is a significant difference between men and women for the relationship between patient dissatisfaction and the number of healthcare providers involved.

In order to calculate the odds ratios for dissatisfaction with the involvement of 0, 1, 2 or ≥3 healthcare providers for women compared to men, we subsequently used a reduced model, excluding the non-significant interaction terms of sex with level of education, duration of the diagnostic pathway, second opinion and experienced delay.

| Variable (*n* = 856)^b^ | Odds ratio (OR) for dissatisfaction | 95% Confidence Interval (CI) | *P* value |
| --- | --- | --- | --- |
| Sex   - Male - Female | Reference  3.11 | 1.4-7.0 | <0.01* |
| Level of education   - Low - Medium - High | Reference  0.41  0.44 | 0.2-0.8  0.2-0.8 | 0.01*  <0.01*  0.01* |
| Duration of the diagnostic pathway   - Unknown - <2 years - ≥ 2 years | Reference  1.19  1.45 | 0.6-2.4  0.8-2.6 | 0.44  0.63  0.20 |
| Number of healthcare providers involved^a^   - 0 - 1 - 2 - ≥3 | Reference  3.59  3.39  4.91 | 1.6-8.1  1.3-8.9  1.3-19.1 | <0.01*  <0.01*  0.01*  0.02* |
| Second opinion   - No/not mentioned - Yes, on the patient’s initiative - Yes, on the healthcare provider’s initiative | Reference  5.26  2.03 | 2.5-11.2  0.8-5.4 | <0.001*  <0.001*  0.16 |
| Experienced delay   - No delay - Not (clearly) mentioned - Yes, caused by the patient - Yes, caused by a healthcare provider - Yes, unknown who caused it | Reference  1.98  0.89  42.18  7.20 | 0.8-5.0  0.2-4.4  21.4-83.2  2.7-19.3 | <0.001*  0.15  0.88  <0.001*  <0.001* |
| Number of healthcare providers involved^a^ x Sex   - 0 x Male - 1 x Male - 2 x Male - ≥3 x Male | Reference  0.19  0.52  0.62 | 0.1-0.6  0.1-2.4  0.1-4.5 | 0.05  <0.01*  0.40  0.64 |

*Statistically significant, P <0.05

^a^Excluding GP and neurologist

^b^Excluding second opinion, initiative unknown

x Sex: interaction term of sex with variable (male = reference group)

Using this reduced model we calculated the odds ratio (OR) for dissatisfaction per number of involved healthcare providers, female compared to male.

| Number of healthcare providers involved^a^ | Odds ratio (OR)  for dissatisfaction, female compared to male | 95% Confidence  Interval (CI) | *P* value |
| --- | --- | --- | --- |
| - 0 | 3.11 | 1.4-7.0 | <0.01* |
| - 1 | 0.58 | 0.2-1.4 | 0.23 |
| - 2 | 1.63 | 0.4-5.8 | 0.45 |
| - ≥3 | 1.92 | 0.3-12.1 | 0.49 |

^a^Excluding GP and neurologist
